# Determination of Glycerol, Propylene Glycol, and Nicotine as the Main Components in Refill Liquids for Electronic Cigarettes

**DOI:** 10.3390/molecules28114425

**Published:** 2023-05-29

**Authors:** Paweł Kubica

**Affiliations:** Department of Analytical Chemistry, Faculty of Chemistry, Gdansk University of Technology, 11/12 Narutowicza Str., 80-233 Gdańsk, Poland; pawkubic@pg.edu.pl

**Keywords:** electronic cigarettes, refill liquids, mass spectrometry, nicotine, liquid chromatography

## Abstract

Refill liquids for electronic cigarettes are an important area of research due to the health safety and quality control of such products. A method was developed for the determination of glycerol, propylene glycol, and nicotine in refill liquids using liquid chromatography, coupled with tandem mass spectrometry (LC-MS/MS) in multiple reaction monitoring (MRM) mode with electrospray ionisation (ESI). Sample preparation was based on a simple dilute-and-shoot approach, with recoveries ranging from 96 to 112% with coefficients of variation < 6.4%. Linearity, limits of detection and quantification (LOD, LOQ), repeatability, and accuracy were determined for the proposed method. The proposed sample preparation and the developed chromatographic method using hydrophilic interaction liquid chromatography (HILIC) were successfully applied for the determination of glycerol, propylene glycol, and nicotine in refill liquid samples. For the first time, the developed method using HILIC-MS/MS has been applied for the determination of the main components of refill liquids in a single analysis. The proposed procedure is rapid and straightforward and is suitable for quick determination of glycerol, propylene glycol, and nicotine. The nicotine concentrations corresponded to the labelling of samples (it varied from <LOD—11.24 mg/mL), and the ratios of propylene glycol to glycerol were also determined.

## 1. Introduction

E-cigarettes are electronic devices that simulate smoking by producing a vapour that is inhaled. They usually contain a battery, a heating element, and a cartridge or tank containing a refill liquid [1]. The refill liquid, liquid solution, or e-liquid usually contain propylene glycol as the main solvent, as well as glycerol, nicotine, flavourings, and other chemicals [2]. However, there are also refill liquids that are called nicotine-free and may contain trace amounts of nicotine up to µg/mL of liquid [3]. The first two ingredients are used to create the vapour that is inhaled and contains nicotine and flavouring. There is a wide range of refill liquids on the market, ranging from traditional tobacco flavours to fruit and dessert flavours [4,5]. However, there are concerns about the safety and regulation of these products, as well as their potential appeal to young people. While these two main ingredients are generally recognised as safe for use in food and cosmetics, there is still debate about their safety when inhaled in the form of vapour [6,7,8,9]. Some studies suggest that they can cause respiratory irritation or other health problems [10,11,12].

Glycerol is a common ingredient in refill liquids that is used with propylene glycol to create the vapour that is inhaled by the user. It is a colourless, odourless liquid commonly used in food and cosmetic products. Propylene glycol has low toxicity and is considered safe for consumption in small amounts. However, as mentioned above, some people may experience allergic reactions or irritation when exposed to high amounts of propylene glycol vapour [13,14]. It is important to know that the safety of refill liquids depends on the ingredients used and the manufacturing process. The nicotine content should also be considered when using refill liquids, as nicotine can be highly addictive and harmful to health. All nicotine used in the production of refill liquids for electronic cigarettes is of natural origin and is obtained from processed parts of tobacco. In addition to the three main ingredients, many different flavour compounds are used to enhance the user’s experience [15,16,17].

There are only a few methods that cover the determination of all three main components of refill liquids. The most common determination methods are based on gas chromatography in combination with different detectors, either flame ionization [18,19,20] or mass spectrometry [21,22]. Some of the techniques for the determination of glycerol, propylene glycol, and nicotine are based on proton nuclear magnetic resonance (^1^HNMR) [23,24,25]. Despite these efforts, to the best of the author’s knowledge, there is no method that uses liquid chromatography coupled with mass spectrometry (LC-MS) or tandem mass spectrometry (LC-MS/MS) in determination of nicotine, propylene glycol, and glycerol. In most cases, sample preparation of refill liquid for the determination of nicotine [2,3,24,26] is based on appropriate dilution and direct injection into the system. A similar procedure is used for the determination of glycerol and propylene glycol [22,26,27]. The simplicity of dilution seems to be a reasonable choice for determining the main components of refill liquids for electronic cigarettes. The liquid is used to produce aerosol by heating it in the tank with the help of heating elements (coils), which are surrounded by cotton wool. The aerosol formed contains nicotine, including the free base and protonated form [28], tobacco-specific nitrosamines, if initially present in the liquid [29], or diacetyl and acetyl propionyl [30]. Other compounds are also found in the aerosol, including toluene, ethyl benzene, ortho-, meta- and para-xylene [31], formaldehyde, acetaldehyde, acetone, and propenal [20,32]. The carbonyl compounds formaldehyde, acetaldehyde, acrolein, propanal, glyoxal, and methylglyoxal were also found in the aerosol formed [33], as well as water vapour, carbon dioxide, and the main components propylene glycol and glycerol [34].

In this paper, a novel method for the determination of nicotine, propylene glycol, and glycerol is presented, in which the dilute-and-shoot approach is used as a sample preparation step, while LC-MS/MS is used for the determination of the analytes. The main idea of the presented method is to propose a simple and accurate approach for the determination of the main components in a single analysis. To the author’s knowledge, the proposed method is the first to use the LC-MS/MS for the determination of nicotine, propylene glycol, and glycerol in refill liquids for electronic cigarettes and without the need of derivatization or complicated sample preparation.

## 2. Results

### 2.1. Detection and Chromatographic Parameters

Separate solutions for each compound were prepared for the determination of glycerol, propylene glycol, nicotine, and two internal standards (ISs): nicotine-d4 and glycerol-d8. All working solutions were prepared in ACN to maintain a concentration of 200 ng/mL for nicotine and nicotine-d4, while the concentrations of glycerol, propylene glycol, and glycerol-d8 were maintained at 2 mg/mL. In preliminary studies, it was decided to use ACN as component B of the mobile phase, determined by the HILIC analysis mode, while component A was initially water. Despite efforts, it was not possible to ionise the glycerol, propylene glycol, and its corresponding ISs. Further studies solved this problem, hence the need to add formic acid (FA) or ammonium formate as buffers to component A of the mobile phase. Optimisation was performed for multiple reaction monitoring (MRM) mode, and the chromatographic system was set to operate in flow injection analysis (FIA) mode. The flow rate during optimisation was 0.25 mL/min, while the composition of the mobile phase was chosen to be 60/40 ACN/20 mM ammonium formate buffer with pH = 4.5. The injection volume was chosen to be 1 µL. In the case of nicotine and nicotine-d4, a simple adduct (M+H)^+^ was observed. In the case of glycerol, propylene glycol, and glycerol-d8, no ion was observed in the positive mode or negative mode of ionisation with water as component A of the mobile phase. Further investigation has shown that formate is required to ensure sufficient ionisation to receive signals coming from these two analytes and their IS. The glycerol, propylene glycol, and glycerol-d8 tend to form the (M+FA-H)^−^ adduct in the negative mode, hence the need to provide the formate in the form of buffer or addition of formic acid to the mobile phase. Further studies proved that the addition of ammonium formate resulted in better separation and better peak shapes compared to the addition of only FA to component A of the mobile phase. For each compound, a specific precursor ion was selected and fragmented in the collision cell, and the two most intense fragments were selected to construct the MRM transition for each analyte and for two ISs with specific collision energy (CE). Finally, to stabilise the signal and improve ionisation for glycerol, propylene glycol, and glycerol-d8, the specific entrance voltages for Q1-Prerod and Q3-Prerod were chosen. For nicotine determination, nicotine-d4 was chosen as its corresponding IS, while, for glycerol and propylene glycol, glycerol-d8 was chosen as their IS.

The proposed dilution (1000×) and optimised transitions resulted in an oversaturated signal for nicotine and its IS when analysing real samples, calibration curve solutions, and fortified samples. In order to be able to analyse, in a single chromatographic run, all three main components of refill liquids for electronic cigarettes, it was decided that CE and entrance voltages Q1 and Q3 for nicotine and its IS should be lowered so as not to produce a very intense signal. Glycerol and propylene glycol did not respond as intensely as nicotine, so the ionisation rate of nicotine and nicotine-d4 had to be lowered. This was performed to avoid separate dilutions of each sample for glycerol and propylene glycol and for nicotine. The lower ionisation rate for nicotine allowed all major components to be analysed with a single dilution and in a single chromatographic run. The parameters of the selected MRM transitions for the analytes and internal standards are listed in Table 1.

**Figure 1:**
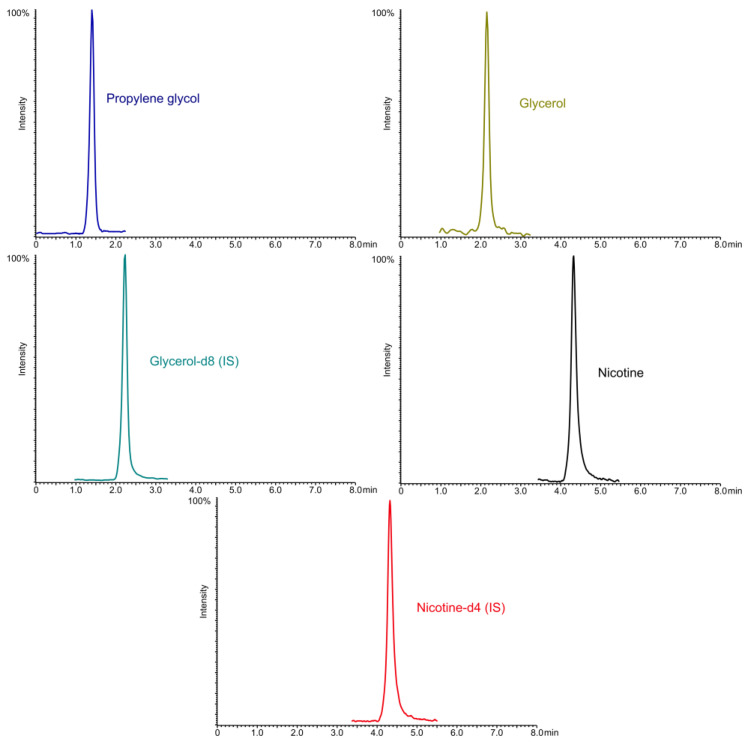
The chromatogram of standards of propylene glycol (2 mg/mL), glycerol (2 mg/mL), nicotine (1 µg/mL), and two corresponding ISs—glycerol-d8 (1 mg/mL) and nicotine-d4 (1 µg/mL).

Finally, all further chromatographic analyses were performed in MRM mode. For the purpose of this study, three different chromatographic columns were used: SeQuant ZIC-HILIC (Merck) 150 × 4.6 mm, 3.5 µm fully porous, Kinetex HILIC (Phenomenex) 100 × 4.6 mm, 3.5 µm fully porous, and Kinetex HILIC (Phenomenex) 100 × 2.1 mm, 1.7 µm with core-shell technology. For all analyses performed with the columns, the mobile phase component B tested was ACN, as determined by the HILIC mode of operation, while the mobile phase component A consisted of water with FA (0.01 to 0.1% of FA) or ammonium formate buffer (5–25 mM) with a pH range of 3.8–6.8. All separations were performed in gradient mode or mixed mode (initially isocratic and gradient), which was simultaneously optimised with temperature, injection volume, and flow rate. Isocratic mode was not considered for all columns tested due to the long retention time of nicotine, as shown by preliminary studies. The SeQuant ZIC-HILIC column did not provide sufficient separation for glycerol and propylene glycol, while the retention time and peak shape of nicotine was not acceptable, despite the conditions used, and propylene glycol and glycerol were observed in the dead time. A similar case was observed with the Kinetex HILIC with fully porous sorbent, despite the separation of glycerol and propylene glycol. The separation of propylene glycol and glycerol was achieved, but the retention time of nicotine and the analysis time (over 10 min) were not within the acceptance criteria. To reduce the retention time, especially for nicotine, and the analysis time, it was decided to use a Kinetex HILIC 100 × 2.1 mm column with core-shell sorbent (1.7 µm). The addition of FA to component A of the mobile phase resulted in poorer peak shapes and lower response compared to the addition of buffer. However, increasing the buffer content above 25 mM resulted in a significant suppression of the signal from propylene glycol and glycerol. To achieve a short separation time (less than 5 min), a sharp gradient of 95% B was chosen after the initial separation in isocratic mode (0–1.2 min), which then dropped to 40% of B (1.2–5 min) and was maintained until 8 min. To achieve a higher signal response for glycerol and propylene glycol, the chromatographic analysis was divided into segments (as shown in Figure 1), while the CE and entrance voltages for nicotine and its IS were minimised to avoid saturation of the detector. Despite the short retention times of propylene glycol and glycerol, it should be noted that the t_0_ for this column is 0.25 min, so that these two substances are separated and, also, as they are the main components of the refill liquids, the probability of interference from other components of the sample is minimised. The chromatograms of separation of nicotine, propylene glycol, and glycerol are presented in the Figure 1.

### 2.2. Sample Preparation Procedure Based on Dilution

An amount of 10 mg of each refill liquid was taken and placed in a 10 mL flask containing ISs (nicotine-d4 and glycerol-d8) at a concentration of 1 mg/mL and 1 µg/mL, respectively. The flask was filled up to the mark with ACN and shaken vigorously for a few minutes to dissolve the refill liquid. The sample dilution was chosen so that 1 mg of liquid to 1 mL of diluted solution was within the concentration range of the calibration curve for glycerol and propylene glycol (0.1–6 mg/mL) and within the concentration range of nicotine (0.05–20 µg/mL). An amount of 1 mL of the diluted sample was injected directly into the LC-MS/MS system. Due to the high viscosity of the refill liquids, the original idea was to pipette samples in reverse mode, but the repeatability of the mass of the refill liquid obtained was not satisfactory (CVs > 10%). Each sample was analysed in triplicate (*n* = 3). The maximum content of nicotine in the refill liquids available on the market is 18 mg/mL, so the correct dilution has been chosen. Most refill liquids on the market usually contain 6 mg/mL or 12 mg/mL of nicotine. In addition, according to market research, there are no refill liquids based only on glycerol, but some are based only on propylene glycol. The most common ratio of propylene glycol and glycerol is 6:4 and 8:2. The content of analytes was recalculated into mg/mL, knowing the weighed mass and density of each refill liquid, including fortified ones. The proposed dilute-and-shoot-based approach proved successful in determining the major constituents of refill liquids.

### 2.3. Method Validation

The performance of the chromatographic method and sample preparation was tested using calibration solutions and prepared fortified samples based on model liquids, containing propylene glycol, glycerol, and nicotine. Three model liquids were prepared: (1) 80% propylene glycol and 20% glycerol with 3 mg/mL nicotine, (2) 60% propylene glycol and 40% glycerol with 6 mg/mL nicotine, and (3) 40% propylene glycol and 60% glycerol with 12 mg/mL nicotine. In this study, the linearity, coefficients of determination (R^2^), slope (a), constant term (b), standard deviation of slope (S_a_), standard deviation of constant term (S_b_), limit of detection (LOD), and limit of determination values were calculated and presented in Table 2. The values of LOD and LOQ are based on the formulas LOD = 3.3S_b_/a and LOQ = 3 × LOD. Glycerol-d8 was used as the IS for glycerol and propylene glycol, while nicotine-d4 was used as the IS for nicotine. To increase the precision of the results obtained in the lower concentration range, the weighted calibration curves were used with a factor of 1/C, where C was the concentration of each calibration solution. Precision based on recovery rates was checked for three model liquids with three replicates each (*n* = 3) and the accuracy as a coefficient of variation (CV) and listed in Table 3. All fortified samples and calibration solutions were prepared according to the protocol described in Section 4.1 and Section 4.3.

The obtained calibration curves were linear in the proposed concentration range, while the R2 values were above 0.9967 for all tested compounds, and the LOD was 0.044 mg/mL for glycerol, 0.039 mg/mL for propylene glycol, and 0.032 µg/mL for nicotine. The recoveries were above 96%, while they did not exceed 112%, and the CVs did not exceed 6.4. The results obtained with the proposed method provide reliable results in terms of repeatability, precision, and accuracy. The method proved useful for determining the main components of refill liquids for electronic cigarettes.

### 2.4. Analysis of Real Samples

Seven samples were bought from the local shop, two denoted as containing 12 mg/mL and these two were the only samples where ratio of propylene glycol to glycerol was labelled (70:30). One sample was labelled as 6 mg/mL of nicotine, two were labelled as 3 mg/mL, and two were labelled as nicotine-free. All samples were chosen based on their popularity and sales ratio. All samples were prepared according to the described protocol and analysed by LC-MS/MS. Obtained results were recalculated to mg/mL, and results are presented in Table 4.

The samples investigated came from different manufacturers. Propylene glycol was found to be the main ingredient in all samples, while glycerol was found in four of them. As already mentioned, there are practically no refill liquids based on glycerine, simply because of its high viscosity and its higher price than propylene glycol. The nicotine content was in some cases lower than declared, which could be due to the fact that part of the nicotine is converted into nicotine N-oxide (all samples were in clear plastic bottles) [35]. Some samples might also contain water that was added intentionally during production or due to the high hygroscopic properties of glycerol and propylene glycol. Samples also contain flavour compounds. However, their content rarely exceeds a few mg/mL, with the exception of menthol, maltol, or ethyl maltol in some samples [16,17].

## 3. Discussion

The developed method was successfully applied for the determination of glycerol, propylene glycol, and nicotine in samples of refill liquids. Presented here, to the best of knowledge, is the first study, focusing on the determination of the three main components of refill liquids using LC-MS/MS. Propylene glycol was detected as the main ingredient of refill liquids in all samples, compared to glycerol, which has a higher viscosity and is more expensive. The nicotine content was as stated on the label. However, nicotine tends to be degraded to nicotine N-oxide in some cases, hence the presumably lower values of this analyte [35]. The simple approach, based on dilution, could be considered an advantage of the presented method. Thus, a lower dilution ratio could be made with respect to glycerol and propylene glycol, whereas a higher dilution should be made for nicotine, which produces an incomparably higher signal in the MS. In this case, it was necessary to reduce the ionisation ratio of nicotine. The other limitations of the proposed method are related to the HILIC separation procedure, so that a long equilibration time of the column is required after each analysis. The low response of glycerol and propylene glycol could be avoided by using a lower dilution. Another disadvantage is the need to check the density of the refill liquids to correctly reflect the results, as most of them are expressed in mg/mL of the liquid sample. The density measurement could be performed with an aerometer or a pycnometer, which are usually cheaper than the former and can also be used with a small amount of sample (5–10 mL).

## 4. Materials and Methods

### 4.1. Standards and Solutions Preparation

Standards for propylene glycol (propane-1,2-diol, CAS: 57-55-6), glycerine (propane-1,2,3-triol, CAS: 56-81-5), nicotine (3-[(2S)-1-methylpyrrolidin-2-yl]pyridine, CAS: 54-11-5) together with two internal standards nicotine-d4 (C_10_H_10_N_2_D_4_, CAS: 350818-69-8), and glycerol-d8 ((DOCD_2_)_2_CDOD, CAS: 7325-17-9) were obtained from Merck (Darmstadt, Germany). All solvents used were of analytical grade (ACN, formic acid) and were purchased from Avantor-VWR (Atlanta, GA, USA), as was ammonium formate. The ultrapure water was obtained using the HLP5 system from Hydrolab (Wiślina, Poland). The separate stock solutions of the analytes and two internal standards were prepared by diluting standards in ACN to obtain 10 µg/mL nicotine and nicotine-d4, 40 mg/mL of propylene glycol, glycerol, and glycerol-d8. The individual stock solutions of analytes and internal standards were further diluted with ACN to obtain working solutions containing 200 ng/mL nicotine and nicotine-d4 and 2 mg/mL propylene glycol, glycerol, and glycerol-d8. The working solutions were used for flow injection analysis (FIA) to optimise detection parameters and chromatographic conditions. Seven calibration solutions were prepared by diluting the working solution to obtain 0.1, 0.5, 1, 1.5, 2, 4, and 6 mg/mL of glycerol and propylene glycol, while the nicotine contents were 0.05, 0.1, 1, 2, 5, 10, and 20 µg/mL. The content of IS was maintained at 1 mg/mL for glycerol-d8 and 1 µg/mL for nicotine-d4 in all calibration solutions. All solutions were stored in the dark in a refrigerator at 4 °C for up to 4 weeks.

### 4.2. Instrument Conditions and Chromatography

The chromatographic system consisted of a LC (Shimadzu, Japan) equipped with a controller (CBM-20A), a degasser (DGU-20A5R), binary pumps (Nexera X2 LC -30 CE), an autosampler (X2 SIL-30AC), and a column oven (CTO 20AC). The chromatographic system was coupled to the Shimadzu LCMS 8060 MS/MS mass spectrometer. All analyses were performed in MRM mode with positive and negative ionization, and the selected parameters of the transitions for each analyte and the two ISs are listed in Table 1. The ion source parameters were maintained as follows: 3 L/min—nebulizing gas flow, 10 L/min—heating gas and drying gas flow, and the temperature of the interface, desolvation line, and heat block were set at 300, 250, and 450 °C, respectively. The capillary voltage was 3.5 kV. The entire system was controlled by LabSolutions software, which was also used for data acquisition and processing. The final optimized method for separation was performed using Kinetex HILIC 100 × 2.1 mm, 1.7 µm (core-shell), 100 Å from Phenomenex (Torrance, CA, USA). The chromatographic conditions after optimization were as follows: component A of the mobile phase: 20 mM ammonium formate buffer pH = 4.5; component B of the mobile phase: ACN. The gradient was chosen as follows: 0–1.2 min 95% B, 1.2–5 min 40% B, 5–8 min 40% B; column stabilization after each analysis was maintained at 95% B for 10 min. The flow rate was maintained at 0.8 mL/min throughout the analysis. The temperature of the separation was set at 45 °C, while the injection volume was chosen to be 1 µL.

### 4.3. Sample Preparation of Refill Liquids and Preparation of Fortified Samples

All seven samples were from different manufacturers, with two samples declared as nicotine-free, two declared as containing 12 mg/mL nicotine, one declared as 6 mg/mL, and two declared as 3 mg/mL. Only two samples were found to have information on the specific ratio of glycerol to propylene glycol (Table 4). From each sample, 10 mg were taken and added to the 10 mL flask together with two ISs and made up to the mark with ACN. The concentration of ISs in the diluted samples was 1 mg/mL glycerol-d8 and 1 µg/mL nicotine-d4. After this dilution, the samples were injected directly into the LC-MS/MS system. Three model liquids were prepared to serve as fortified samples. The model liquids were prepared by mixing glycerol and propylene glycol to obtain (1) 80% propylene glycol and 20% glycerol with 3 mg/mL nicotine, (2) 60% propylene glycol and 40% glycerol with 6 mg/mL nicotine, and (3) 40% propylene glycol and 60% glycerol with 12 mg/mL nicotine. The fortified samples were subjected to the same sample preparation procedure as the real samples.

## 5. Future Perspectives

The proposed studies will provide better insight into the composition of refill liquids and improve the quality control of such products. The next steps should address emission profiles, as it has been found that different compositions of refill fluids produce different aerosols [36]. In addition, different ratios of propylene glycol to glycerol can lead to the formation of different products during aerosolization [11,37]. The next step should be to apply the mass change tracking ratio (MCT) during the production of aerosol from refill liquid with an automatic smoker/vaporiser and determine the influence of the generation temperature or applied power. The author believes that the proposed method can be successfully applied for the rapid determination of the three main constituents of refill liquids in a single run.

## Figures and Tables

**Table 1:** Parameters of chosen MRM transitions for selected analytes.

Analyte	Precursor Ion Ionization Type	Product Ions	Collision Energy [V]	Q1 Prerod [V]	Q3 Prerod [V]
Glycerol	137.1 (M+FA-H)^−^	45.0 ^1^ 91.0	10 15	25 25	11 11
Propylene glycol	121.2 (M+FA-H)^−^	45.0 ^1^ 77.1	8 14	18 17	10 11
Nicotine	163.1 (M+H)^+^	117.1 ^1^ 130.1	26 19	12 12	24 24
Glycerol-d8 (IS)	142.1 (M+FA-H)^−^	45.0 ^1^ 142.1 ^2^	10 5	16 16	16 16
Nicotine-d4 (IS)	167.1 (M+H)^+^	121.1 ^1^ 134.1	27 21	11 11	26 26

^1^ Quantitative transition. ^2^ Pseudotransition was chosen as confirmation one.

**Table 2:** Parameters of calibration curves, including S_a_, S_b_, LOD, LOQ, and R^2^.

Analyte	Equation of Calibration Curve	S_a_	S_b_	LOD	LOQ	R^2^
Glycerol	y = 1.500x + 0.105	0.020	0.020	0.044 mg/mL	0.13 mg/mL	0.9975
Propylene glycol	y = 0.01897x − 0.00073	0.00022	0.00023	0.039 mg/mL	0.12 mg/mL	0.9991
Nicotine	y = 4.014x − 0.060	0.035	0.039	0.032 µg/mL	0.095 µg/mL	0.9967

**Table 3:** Recoveries, together with SDs and CVs, for prepared model liquids. All values were recalculated according to the weighed mass of the sample and the known density of the model liquid.

Analyte	Fortified Level Based on Model Liquid	Recovery mg/mL (%)	SD	CV [%]
Glycerol	60%	631 (105)	11	3.5
	40%	425 (106)	13	3.3
	20%	224 (112)	10	6.4
Propylene glycol	80%	830 (104)	13	1.5
	60%	626 (104)	14	2.3
	40%	425 (106)	11	2.8
Nicotine	3 mg/mL	3.15 (105)	0.13	4.2
	6 mg/mL	5.76 (96)	0.14	2.4
	12 mg/mL	11.67 (97)	0.18	1.6

**Table 4:** Determined concentrations of glycerol, propylene glycol, and nicotine in the real samples of refill liquids for electronic cigarettes.

Sample	Declared Nicotine Content (mg/mL)	Declared Ratio of Propylene Glycol to Glycerol	Glycerol ± SD (mg/mL)	Propylene Glycol ± SD (mg/mL)	Nicotine ± SD (mg/mL)
1. Ice Mint	12	70:30	272.3 ± 7.8	722 ± 16	10.66 ± 0.12
2. Grape	12	70:30	261.7 ± 8.0	752 ± 13	11.24 ± 0.11
3. Bubble-gum	6	-	224.0 ± 9.5	830 ± 10	5.85 ± 0.10
4. Pineapple and Maracuja	3	-	<LOD	1073 ± 24	3.135 ± 0.084
5. Desert Ship	3	-	<LOD	1086 ± 21	2.914 ± 0.094
6. Tobacco	0	-	321.7 ± 7.0	732 ± 17	<LOD
7. Strawberry	0	-	<LOD	1046 ± 18	<LOD

## Data Availability

The data presented in this study are available upon request from the corresponding author.
